# Another Aspirant for Veterinary Fame—Fools Rush in Where Angels Fear to Tread

**Published:** 1898-06

**Authors:** 


### ANOTHER ASPIRANT FOR VETERINARY FAME—FOOLS RUSH IN WHERE ANGELS FEAR TO TREAD.

S——, INDIANA, May 23, 1898.

SIR: What Would a Catalogue Cost me. Givin Pr of Veterinary instments and Case for Medicine and Sergen instement i Have read Medicine and as a man Who live in the country Have Had lot of Experince

Also What Would one of the Best Veterinary Book. Cost me. i am Satisfied that Withe the proper. instment and proper meidicine i can Head off lots of the Deses am Well pleas with your work. Can give Best of Refference

Remain your truly
Write
S A—
S——, Indiana

